# Lyme Carditis: A Rare Presentation of Sinus Bradycardia Without Any Conduction Defects

**DOI:** 10.7759/cureus.5554

**Published:** 2019-09-02

**Authors:** Brittney A Grella, Mihir Patel, Satish Tadepalli, Christopher W Bader, Kenneth Kronhaus

**Affiliations:** 1 Family Medicine, Hackensack Meridian Health, Ocean Medical Center, Brick, USA; 2 Internal Medicine, Hackensack Meridian Health, Ocean Medical Center, Brick, USA

**Keywords:** lyme carditis, lyme disease, sinus bradycardia, bradycardia

## Abstract

Lyme carditis is a rare cardiac manifestation of Lyme disease that occurs when bacterial spirochetes infect the pericardium or myocardium triggering an inflammatory response. The most common electrocardiogram (EKG) findings in these patients include atrioventricular (AV) conduction abnormalities (first, second, and third degree heart block).

A 56-year-old male with a history of hypothyroidism, from the Northeastern region of the United States, presented to the emergency department with lightheadedness and chest pain. His EKG revealed sinus bradycardia with a heart rate of 49 beats per minute, without ST segment elevation, T wave inversions, or signs of heart block. An enzyme-linked immunosorbent assay (ELISA) Lyme titer was elevated, and confirmatory Western blot was positive for IgG and negative for IgM. He was treated with intravenous (IV) ceftriaxone; however, he continued to have persistent bradycardia with his heart rate dropping to 20 to 30 beats per minute throughout the night. Additionally, he had several sinus pauses while sleeping, with the longest lasting for 6.1 seconds. A pacemaker and an additional three-week course of IV ceftriaxone was determined to be the best treatment for his resistant bradycardia secondary to Lyme carditis. No symptoms were present at his one month follow-up appointment, as an outpatient, after completing ceftriaxone therapy. The patient follows up with cardiology regularly to have his pacemaker checked.

Here we present a unique case of Lyme carditis, without the classical findings of Lyme disease or common EKG findings of AV conduction abnormalities. A high clinical suspicion of Lyme carditis is required when someone from a Lyme endemic region presents with unexplained cardiac symptoms and electrocardiogram abnormalities. This case report aims to add to the knowledge gap between suspicion of Lyme carditis and sinus bradycardia as the only presenting symptom.

## Introduction

Lyme carditis is a rare cardiac manifestation of Lyme disease that can be fatal if left untreated. An estimated 4%-10% of untreated Lyme disease cases progress to Lyme carditis, and in the United States 1.5%-10% of untreated Lyme disease presents as Lyme carditis. The actual incidence of Lyme carditis is unknown; the carditis usually goes undiagnosed because heart biopsies are rarely performed [1].

Previous studies suggest that only up to 50% of cases present with the characteristic erythema migrans rash [2]. The postulated mechanism is that bacterial spirochetes (*Borrelia burgdorferi *or* Borrelia mayonii*) infect the pericardium or the myocardium, triggering an inflammatory response, which results in Lyme carditis [3]. Although only a few cases of untreated Lyme disease develop cardiac manifestations, Lyme carditis is notorious for causing sudden cardiac death. Therefore, a high clinical suspicion of Lyme carditis is required when someone from a Lyme endemic region presents with unexplained cardiac symptoms and has EKG findings suggestive of carditis.

Here we present a case of a 56-year-old male who presented with lightheadedness, chest pain, and bradycardia.

## Case presentation

A 56-year-old male with a history of hypothyroidism, from the Northeastern region of the United States, presented to the emergency department with lightheadedness, chest pain, and bradycardia with a heart rate of 33 beats per minute. On the morning of admission, the patient walked to the bathroom and began experiencing intermittent left-sided chest pain prompting him to come to the hospital. The chest pain was rated 2 out of 10 in intensity, was localized to the left side, was described as a heavy sensation, and was non-radiating. The patient denied having shortness of breath, cough, nausea, vomiting, or diaphoresis. He never experienced previous episodes of chest pain on exertion or at rest. A few weeks prior to admission, the patient began experiencing occasional mild headaches and lightheadedness upon standing and walking. He began taking 50 mcg of levothyroxine four months ago. He did not have a family history of sudden cardiac death or coronary artery disease. He denied any history of smoking, alcohol, or drug use. He denied any recent travel. A ten-point review of systems was completed and negative except as above.

**Figure 1:**
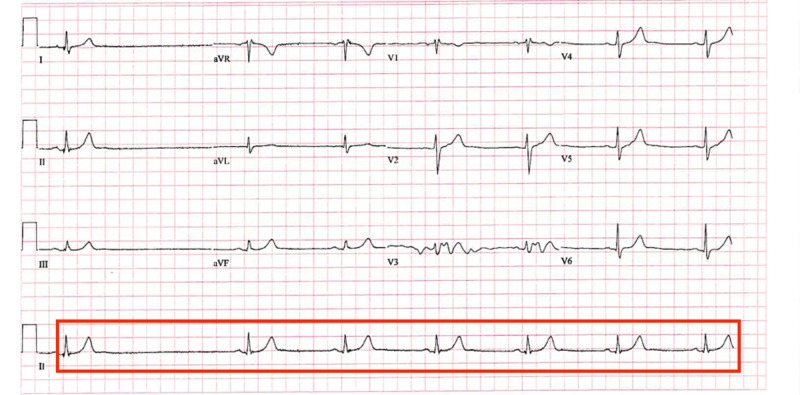
Patient’s electrocardiogram (EKG) revealing a 1.8 second sinus pause and sinus bradycardia without any atrioventricular (AV) conduction defects.

On presentation, his vitals were as follows: temperature 98.2˚F, a pulse of 33 beats per minute, respiratory rate of 18 breaths per minute, blood pressure of 131/71 mmHg, and oxygen saturation of 97% on room air. On exam, the patient was in no acute distress. He had no jugular vein distention (JVD) or carotid bruits. His S1 and S2 heart sounds were normal and no murmurs were heard on auscultation. The remainder of the physical exam was within normal limits. His laboratory results were as follows: first troponin of 0, a second troponin (six hours after the first troponin) of < 0.01, a third troponin (six hours after the second troponin) of < 0.01, a neutrophil percentage of 44.9 (50% -70%), lymphocyte percentage of 43.4 (25% - 43%), monocyte percentage of 9.3 (0.0% - 0.9%), free T4 of 0.93 ng/dl (0.5 - 1.26 ng/dl ), and TSH of 1.188 IU/ml (0.3 - 4.5 IU/ml). A lipid panel showed a high-density lipoprotein (HDL) level of 34 (39 - 79 mg/dl), very low-density lipoproteins (VLDL) of 29 (<20 mg/dl), and low-density lipoprotein (LDL) of 130 (<130mg/dl). His EKG revealed sinus bradycardia with a heart rate of 49 beats per minute and a 1.8 second sinus pause (Figure 1). The PR interval was within normal limits. There were no dropped beats, ST segment elevations, or T wave inversions.

His chest X-ray showed no acute pulmonary disease. A transthoracic echocardiogram was performed, which showed a mildly reduced left ventricular systolic function with an ejection fraction of 51%-54%. As there was a low probability of acute coronary syndrome in this patient with negative cardiac enzymes and no suggestive EKG changes, ST-elevation myocardial infarction and non-ST-elevation myocardial infarction were ruled out. Considering that the patient had unexplained cardiac symptoms, with persistent bradycardia, normal thyroid function studies, and lives in a Lyme endemic region, an ELISA Lyme titer was obtained and found to be elevated at 2.89 (0-0.9). A confirmatory Lyme Western blot was completed, which showed the patient was positive for IgG (positive bands included: 18, 28, 39, 41, 45, 58, 66, 93) and negative for IgM (band 23 was positive), based on the Centers for Disease Control and Prevention (CDC) criteria [4].

At this point, a detailed retrospective history from the patient unveiled that he remembered having ticks crawling on him. However, he denied any noticeable insect bites, fever, rash, and joint pains. Infectious disease was consulted, and it was presumed that the patient had Lyme carditis and was treated with intravenous (IV) ceftriaxone for the next seven days. His heart rate remained consistently low, dropping to 20-30 beats per minute throughout the night with several sinus pauses while sleeping, the longest having lasted for 6.1 seconds. It was determined that a pacemaker would be the preferred choice of treatment for his resistant bradycardia secondary to Lyme carditis. The pacemaker was placed and the patient received an additional three-week course of ceftriaxone therapy. The patient was discharged and asked to follow-up with his primary care physician, cardiology, and infectious disease as an outpatient. At his follow-up appointment, no symptoms were reported. He denied having chest pain, lightheadedness, fever, or joint pains. His heart rate was stable at 62 beats per minute. The patient completed the one-month course of antibiotics and is following up with cardiology regularly to check his pacemaker.

## Discussion

In 1980, Steer et al. first described Lyme carditis in a case series of 20 patients who presented with fluctuating degrees of heart block [3]. In addition to having heart block, the patients also presented with symptoms that mimicked acute rheumatic fever. These patients were ultimately found to have Lyme carditis, a tick-borne disease caused by infection of the heart muscle by the spirochete bacteria *Borrelia burgdorferi and Borrelia mayonii*. The study showed that Lyme carditis usually develops two to three months after an untreated tick bite [5].

According to Fish et al., Lyme carditis is commonly seen between the months of June and December. Though 4%-10% of untreated Lyme disease progresses to Lyme carditis, an estimated 30% have asymptomatic carditis [1].

Clinical manifestations

Patients with Lyme disease may present with the characteristic erythema migrans rash and flu-like symptoms including fever, headache, myalgias, and arthralgias. If left untreated, cardiac manifestations can develop within 21 days of onset of erythema migrans. Approximately 4%-10% of people infected with Lyme disease develop symptoms from cardiac involvement. A review of Lyme carditis cases carried out by the CDC revealed that the patients reported the following symptoms: palpitations (69%), conduction abnormalities (19%), myocarditis (10%), left ventricular systolic dysfunction (5%), and hospitalization (21%) [1].

The typical clinical manifestation of Lyme carditis is attributed to a self-limiting AV conduction abnormality that presents as dizziness, syncope, and shortness of breath with or without chest pain [6]. Atrioventricular blocks can vary from a first-degree heart block to a fatal third-degree heart block or asystole. The spirochete can affect any cardiac muscle layer and the patient may present with pericardial effusion, congestive heart failure, tachyarrhythmia, or QT prolongation. Approximately 35% of patients present with bradycardia and 15% present with tachycardia. Other common cardiac manifestations include myopericarditis, intraventricular conduction disturbances, and bundle branch blocks. Patients who develop Lyme carditis can present with subtle symptoms including lightheadedness, fainting, palpitations, chest pain, and shortness of breath. On physical examination, it is common to have bradycardia, signs of congestive heart failure with canon “a” waves in jugular venous pressure, erythema migrans, monoarthritis, and sometimes central nervous system manifestations including meningoencephalitis, cranial nerve palsies, etc.

Serology 

Determining if a patient has Lyme disease requires serologic testing. First, an enzyme-linked immunosorbent assay (ELISA) or enzyme-linked fluorescent immunoassay (ELFA) is performed [7]. Presence of IgM antibodies on ELISA indicates an active infection that has been present for up to four weeks. Presence of IgG antibodies on ELISA indicates a chronic infection that has been present for a more extended period (four to six weeks). It is important to note that there may be false positives, especially because ELISA can be positive in other medical conditions.

Next, Lyme disease is confirmed using a Western Blot [4]. This is a highly specific test used for the diagnosis of Lyme disease. Although serological testing initially can give false negative results, repeat serology must be performed within two to six weeks in a patient with a strong suspicion of Lyme carditis [8].

According to the CDC, a positive IgM Western blot occurs when at least two of three bands are positive (21-24, 39, and 41kDa) and a positive IgG Western blot occurs when at least five of 10 bands are positive (18, 21-24, 28, 30, 39, 41, 45, 58, 66, and 93kDa). According to German Borreliosis Society guidelines, the presence of any one of following highly specific antibodies is interpreted as Lyme disease: p18, p21, p22, p23, p24, p25, p39, p58 (Tables 1-2).

**Table 1:** CDC interpretation and diagnostic criteria for Lyme disease. CDC - Centers for Disease Control and Prevention.

CDC Western Blot Criteria		
Positive IgM	2 of the following 3 bands are present: 24 kDa (OspC) 39 kDa (BmpA) 41 kDa (Fla)	A positive IgM is sufficient to diagnose in early Lyme disease (< 4 weeks of onset of symptoms).
Positive IgG	5 of the following 10 bands are present: 18 kDa 21 kDa (OspC) 28 kDa 30 kDa 39 kDa (BmpA) 41 kDa (Fla) 45 kDa 58 kDa (not GroEL) 66 kDa 93 kDa	At any point in infection, a positive IgG is diagnostic of Lyme disease.

**Table 2:** Antigen specificity in Western blot (Deutsche Borreliose-Gesellschaft e.v., 2010)*. * Refer [2].

Borrelia Antigen	Specificity
p18	High
p21	High
p22, 23, 24, 25	High
p39	High
p41	Unspecific
p58	High
p66	Unspecific

However, it is crucial to keep in mind not to interpret the presence of fewer bands as a positive serology in Lyme disease, as antibodies to several borrelial antigens are cross-reactive with non-borrelial antigens. Therefore, having less than two IgM bands or less than four IgG bands are not indicative of Lyme carditis by CDC criteria.

Diagnostic criteria for Lyme carditis

Below are the diagnostic criteria for Lyme carditis [9].

1. Clinical picture: New AV conduction defects or arrhythmias. Signs and symptoms of perimyocarditis, history of tick bite and erythema chronicum migrans.

2. Laboratory testing:Borrelia burgdorferi antibodies in serum.

3. Specific testing: Myocardial biopsy - presence of Borrelia DNA in the polymerase chain reaction (PCR)

**Figure 2:**
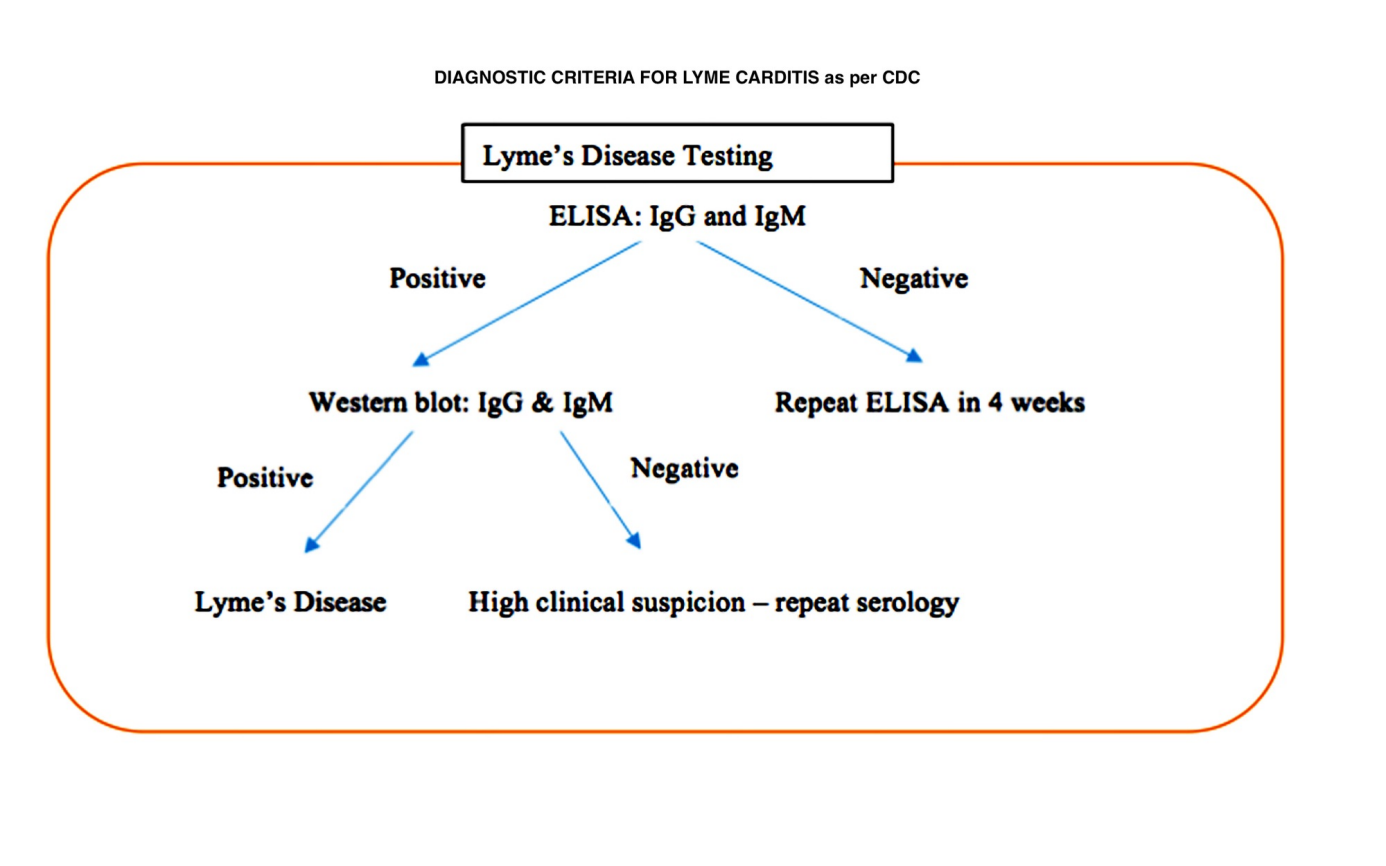
CDC's diagnostic criteria for Lyme disease. CDC - Centers for Disease Control and Prevention.

Below are the diagnostic criteria for Lyme carditis as per the CDC (Figure 2).

The suspicious index in Lyme carditis (SILC) score

In a recent article, Besant et al. proposed a new scoring system after retrospectively reviewing 84 cases of confirmed Lyme carditis (Table 3). The suspicious index in Lyme carditis (SILC) score was developed to evaluate the possibility that a patient's high‐degree heart block can be attributed to Lyme carditis. A SILC score of 0-1 is low, 3-6 is intermediate, and 7-12 is high suspicion for Lyme carditis. The sensitivity of SILC risk of Lyme carditis is 93.2%, but no specificity was reported. Our patient’s score was four (male, endemic area, presyncope), which puts him under intermediate risk of Lyme carditis [10, 11].

**Table 3:** The suspicious index in Lyme carditis scoring system as proposed by Besant et al.*. *Refer [11].

Patient's Characteristics	Score
Age < 50	1
Male	1
Outdoor activity/endemic area	1
Constitutional symptoms (malaise, fever, arthralgias, dyspnea, pre syncope and syncope)	2
Tick bite	3
Erythema migrans	4

Histopathology

Endomyocardial biopsy is the gold standard used to diagnosis Lyme carditis [12]. A typical biopsy specimen will show characteristic transmural inflammatory infiltrates, with a band-like endocardial lymphocyte infiltration [9]. Occasionally the spirochete can be found within the muscle fibers, within the myocardium, or in the inflammatory infiltrates. Polymerized chain reaction (PCR) of the endomyocardial biopsy specimen can also establish the diagnosis of Lyme carditis, but a negative PCR does not exclude Lyme carditis.

The possible mechanism for cardiac damage in Lyme carditis is attributed partly to the inflammatory reaction induced by the spirochete bacteria in addition to the direct cytotoxic effect of the spirochete itself.

The original understanding of the pathogenesis of Lyme carditis has been through rat models. Spirochete inoculated mice show peak inflammatory changes in the first two to three weeks of infection. After a month of inoculation, inflammatory infiltrates were found in the aortic root, the base of the heart, the atrial and ventricular epicardium, the endocardium, the myocardium, and in perivascular spaces especially the AV junction. Unlike neutrophil predominance as seen in Lyme arthritis, macrophage predominance is the typical histopathological picture seen in murine Lyme cardiac models. Immunofluorescence technique can be used to demonstrate the presence of spirochete in the cardiac muscle [1, 13].

EKG

According to Steer et al., a 12-lead EKG and Holter monitor can show ST-segment depression, T-wave inversions in inferolateral leads, and AV conduction abnormalities [5, 9]. However, the most common findings are AV conduction abnormalities. First degree AV block was seen in 90% of patients, and complete heart block was observed in 44% of patients. A progression of first-degree AV block to complete AV block was very common when the PQ interval was > 300 msec. Our literature search revealed that 87% of patients with Lyme carditis developed a first degree AV block, and 53% of patients had a complete AV block or Mobitz type block.

In our literature review, we found that very few cases of Lyme carditis have undergone electrophysiological studies as a part of the workup for Lyme carditis [12]. The cases that have undergone electrophysiological studies showed that heart block (AV block) occurred above the bundle of His and at the same time simultaneous multiple blocks were also noted in SA node, atrium, bundle branches, and fascicles. Furthermore, Lyme carditis patients can also present with a sino-atrial block, temporary bundle block, paroxysmal atrial fibrillation, and prolonged QT interval. AV block development in Lyme carditis patents is attributed to the host's immune response to B. burgdorferi infection of the myocardial tissue.

Imaging

Echocardiography can provide valuable information on wall motion abnormalities [9]. Though echocardiographic findings are not explicit for Lyme carditis, echocardiography is an excellent diagnostic tool for evaluating the presence and degree of cardiac dysfunction and therefore can provide essential information for the management of these patients. Left ventricular wall motion dysfunction, especially abnormal ventricular wall kinetics, on echocardiogram can indicate myocarditis.

Cardiac MRI is another diagnostic modality that can provide valuable information about pericardial involvement [14]. For example, wall edema, which presents as increased signal density on T1 weighted images, corresponds to the inflammatory process triggered by the spirochete.

Treatment

A gray zone exists in regards to the treatment of Lyme disease because every patient does not present with the characteristic rash and symptoms of Lyme disease. Consequently, previous studies and their results cannot be generalized due to the high variability of clinical presentation and population heterogeneity [15].

There are two separate guidelines put forth in regards to Lyme disease treatment. The Infectious Diseases Society of America (IDSA) recommends that patients be started on a short course of antibiotics as the persistent infection is infrequent or non-existent [16]. However, the International Lyme and Associated Diseases Society (ILADS) recommends the Grading of Recommendations, Assessment, Development, and Evaluation (GRADE) system. This system emphasizes that a prolonged course of antibiotics be required, keeping in mind the high failure rates from a short course of antibiotics and the high prevalence of disseminated disease in a large number of cases.

Treatment of Lyme carditis is principally based on the severity of conduction disease [17]. Patients with Lyme carditis who do not have high-grade heart block are managed conservatively with oral antibiotics. The duration of antibiotic management depends primarily on adequate shortening of the PR interval and the normalization of AV block.

On the other hand, patients with a high-grade heart block should be hospitalized [16], closely monitored, and treated with IV ceftriaxone 2 grams or IV penicillins for second/third-degree AV block or prolonged for PR interval > 300 ms. As per the European Federation of Neurological Societies (EFNS), ceftriaxone or cefotaxime for two weeks is the recommended standard of care in acute Lyme carditis [18].

Although many Lyme carditis patients often recover well within a week of antibiotic treatment, it is often recommended to treat acute cases of Lyme carditis with a two-week course of ceftriaxone to eliminate the spirochete from blood and heart muscle [19]. Temporary pacing may be required in patients with an advanced degree of heart block who cannot be managed conservatively.

The following antibiotic regimens are recommended by the CDC, ILADS, IDSA, and German Borreliosis Society (Tables 4-5).

**Table 4:** Antibiotic regimens recommended by the CDC, ILADS, and IDSA for the treatment of Lyme carditis. CDC - Centers for Disease Control and Prevention; ILADS - International Lyme and Associated Diseases Society; IDSA - Infectious Disease Society of America.

Lyme Carditis Treatment Regimens	
Centers for Disease Control and Prevention (CDC)	
Doxycycline 100 mg orally twice daily for 10-21 days or Cefuroxime Axetil 500 mg orally twice daily for 14-21 days or Amoxicillin 500 mg orally three times daily for 14-21 days	
International Lyme and Associated Diseases Society (ILADS)	
Amoxicillin 1500-2000 mg orally daily in divided doses for 4-6 weeks or Cefuroxime 500 mg orally twice daily for 4-6 weeks or Doxycycline 100 mg orally twice daily for for 4-6 weeks or Azithromycin 250-500 mg orally daily for 21 days	
Infectious Disease Society of America (IDSA)	
Preferred Treatment	Ceftriaxone 2 grams once per day via IV for 14 days with a range of 10-28 days
Alternative Treatments	Cefotaxime 2 grams IV every 8 hours or Penicillin G 18-24 million units per day in patients with normal renal function divided into doses given every 4 hours or Doxycycline 200-400 mg per day in 2 divided doses orally for 10-28 days for patients intolerant of B-lactam antibiotics

**Table 5:** German Borreliosis Society recommendations for the treatment of Lyme disease.

Antibiotics applicable in Lyme disease (Deutsche Borreliose-Gesellschaft e.v., 2010) [2]
Beta lactams: Ceftriaxone, Cefotaxime, Cefuroxime Axetil, Benzathine benzylpenicillin, Phenylmethyl Penicillin, Amoxicillin
Tetracyclines and glycylcyclines: Doxycycline, Minocycline
Macrolides: Clarithromycin, Azithromycin
Nitromidazoles: Metronidazole
Co-drugs: Hydroxychloroquine

Prognosis

There should be a high clinical suspicion of Lyme carditis in patients with unexplained syncopal episodes, dyspnea, and chest pain, who live in a region where Lyme disease is prevalent [19]. Patients with second- or third-degree AV block or first-degree AV block with a prolonged PR > 300 ms should be admitted and closely monitored because these patients can deteriorate rapidly [20]. In almost 35% of cases, patients require temporary heart pacing. However, heart block associated with Lyme carditis resolves with antibiotics and seldom requires a permanent pacemaker. Typically, after one to five weeks of treatment one can expect resolution of symptoms.

Several studies funded by the National Institutes of Health (NIH) on the treatment of Lyme disease have shown that most people recover well within a few weeks of initiation of oral antibiotics. A few cases have reported mortality from sudden cardiac death due to lymphocytic infiltration of the heart [5, 6]. However, if prompt admission and antibiotic treatment are initiated, the overall prognosis of Lyme carditis is good.

## Conclusions

Here we present a unique presentation of Lyme carditis, without any classical findings of Lyme disease such as rash or any common EKG findings of heart block. This case report adds knowledge to the gap between suspicion of Lyme carditis and sinus bradycardia as the only presenting symptom.
